# Supplementary material for: Insulin-activated store-operated Ca2+ entry via Orai1 induces podocyte actin remodeling and causes proteinuria
Source: Nat Commun. 2021 Nov 11;12:6537. doi: 10.1038/s41467-021-26900-w (PMC8586150; doi:10.1038/s41467-021-26900-w)
Supplement: Supplementary file 1 — Supplementary Information [file 41467_2021_26900_MOESM1_ESM.pdf]

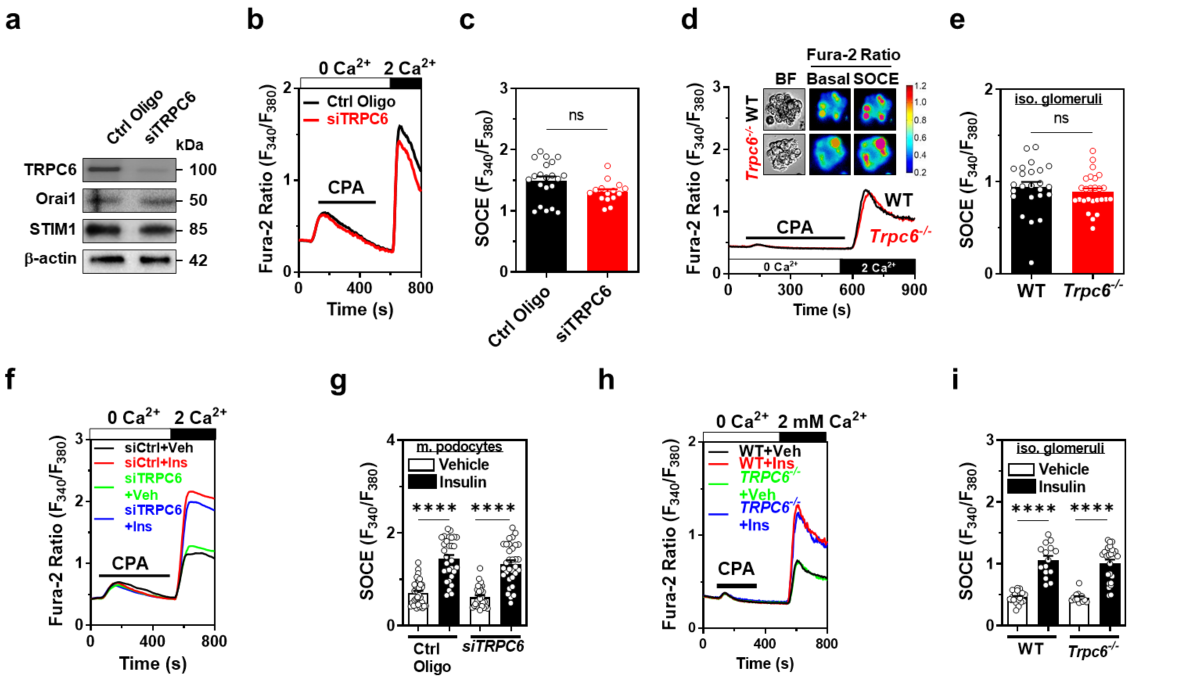

Supplementary Fig. 1 TRPC6 may not participate in store-operated Ca^{2+} entry (SOCE) in podocyte.

a Effect of TRPC6 siRNA knockdown on the expression of Orai1 and STIM1 in mouse podocytes. Repeated 3 independent experiments. **b** Representative traces of fura-2 ratio representing the effect of TRPC6 silencing on SOCE in podocytes. **c** Summary of the SOCE in panel (**b**). *n* = 21 and 15 cells, respectively. **d** Representative fluorescence images (upper panel) and traces of fura-2 ratio (lower panel) in acutely isolated mouse glomeruli from wild type (WT) and *Trpc6*$^{-/-}$ mice. **e** Summary of the SOCE in panel (**d**). *n* = 24 (WT), and 26 (*Trpc6*$^{-/-}$) glomeruli. **f** Representative SOCE traces showing the effect of TRPC6 knockdown on insulin-stimulated SOCE in cultured mouse podocytes. **g** Summary of the SOCE in panel (**f**). *n* = 37, 30, 28, 32 cells per each group. **h** Representative SOCE traces showing insulin (100 nM, 1 h)-activated SOCE in acutely isolated mouse glomeruli from WT and *Trpc6*$^{-/-}$ mice. **i** Summary of the SOCE in panel (**h**). *n* = 21, 15, 14, and 24 glomeruli each. Data are expressed as mean ± SEM analyzed with unpaired two-tailed Student's *t*-test to Ctrl Oligo and WT (**c** and **e**) and two-way ANOVA followed by Tukey's multiple comparisons test (**g** and **i**). ****$p < 0.0001$, ns., not significant.

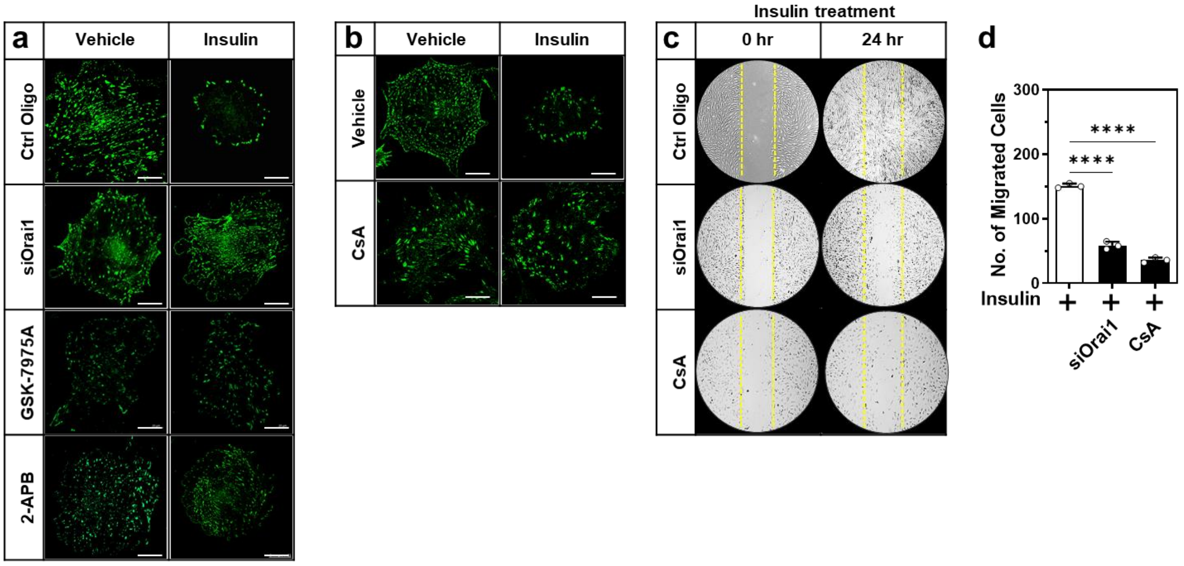

Supplementary Fig. 2 Insulin stimulation of Orai1 promotes disruption of focal adhesion and cell migration.

a Effect of Orai1 inhibition by siRNA knockdown or its blockers, GSK-7975A (3 μM) or 2-APB (100 μM), on insulin-induced redistribution of paxillin in cultured mouse podocytes. **b** Effect of cyclosporine A (CsA; 10 μM) on insulin-mediated redistribution of paxillin in cultured mouse podocytes. Scale bar = 50 μm., All images were represented similarly from 3 independent experiments and randomly chosen10~12 cells in each dish (**a** and **b**). **c** Scratch assay showing the effect of siRNA Orai1 (siOrai1) and CsA on insulin-stimulated podocyte motility. **d** Cell motility was evaluated by counting the number of migrated cells into the wound area after 24 h. *n* = 3 independent experiments. Data are expressed as mean ± SEM and analyzed with one-way ANOVA followed by Dunnett's multiple comparisons test (**d**). ****p < 0.0001.

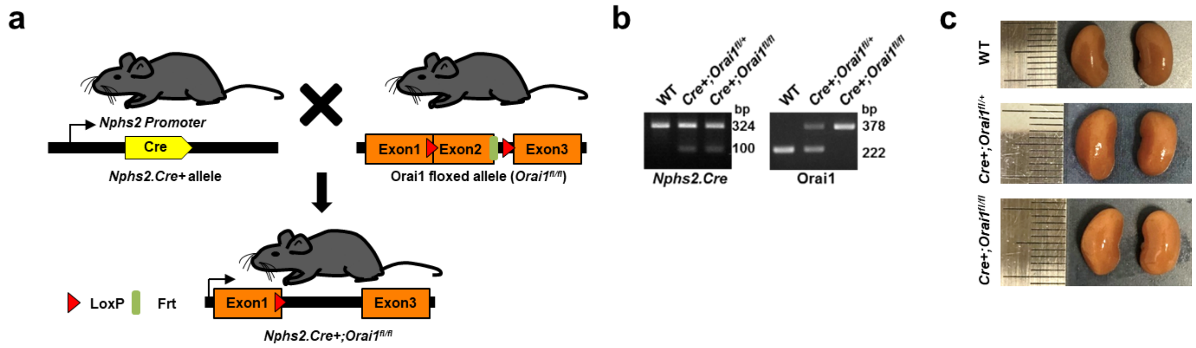

Supplementary Fig. 3 Construction and macroscopic kidneys of podocyte-specific *Orai1*-deletion mice.

a Schematic representation of the *Nphs2.Cre+* (upper left) and *Orai1*$^{fl/+}$ (upper right) mice to generate podocyte-specific Orai1 knockout. Exon 2 of the Orai1 gene was targeted[46] in the *Nphs2.Cre+;Orai1*$^{fl/fl}$ mice (lower). **b** Germ-line transmission of Cre and flox alleles was validated by genotyping PCR. The 324-base pair (bp) band is the international positive control and the 100 bp band denotes *Nphs2.Cre+*(left). The 378-base pair (bp) band is derived from the floxed allele while the 222-bp band denotes the wild-type (WT) allele (right). **c** No difference in kidney size was observed between podocyte-specific Orai1 knockout and WT mice at 10 weeks. Scale bar = 1 mm.

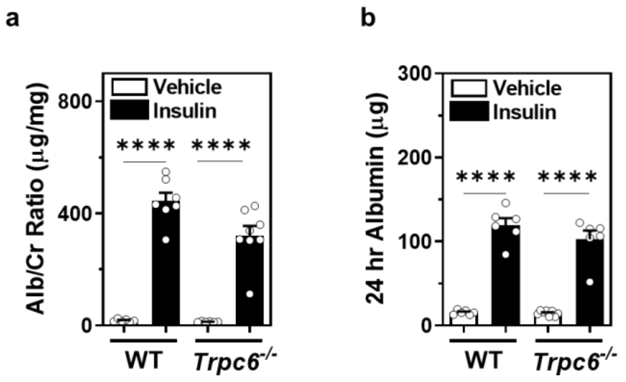

Supplementary Fig. 4 TRPC6 may not participate in insulin-stimulated proteinuria.

a-**b** Quantitative analysis of 24 h urinary albumin/creatinine (Alb/Cr) ratio (left) (**a**) and albumin excretion (right) (**b**) after administration of insulin (*i.p.;* 5 U/kg) in wild type (WT) and *Trpc6*$^{-/-}$ mice (9-11 weeks old, n = 6, 7, 6, 8 mice each). Data are represented as mean ± SEM and were analyzed using analyzed with two-way ANOVA followed by Tukey's multiple comparisons test (**a** and **b**). ****$p < 0.0001$.

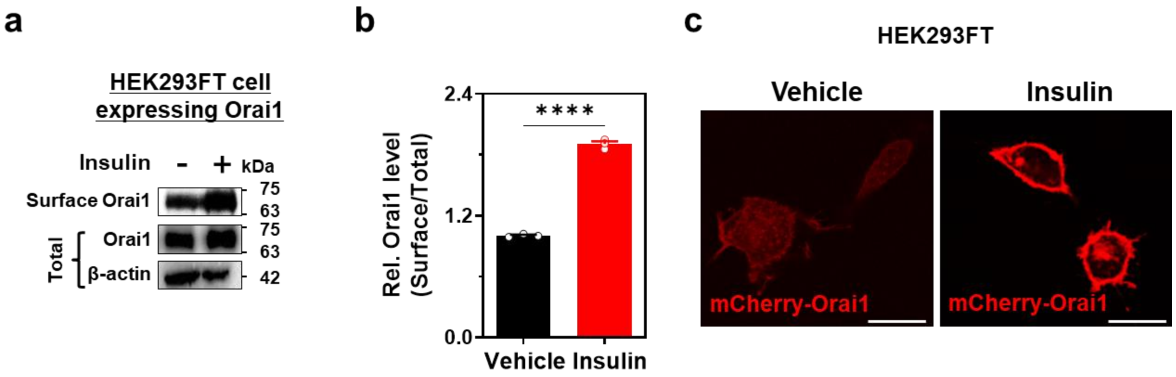

Supplementary Fig. 5 Insulin increases cell surface abundance of Orai1 in HEK293FT cells

a Biotinylation assay showing the insulin effect (100 nM, 1 h) on the cell-surface abundance of Orai1 in HEK293FT cells transiently expressing mCherry-Flag-tagged Orai1 and YFP-tagged STIM1. **b** Quantification of surface Orai1 by densitometry in three independent experiments of panel (**a**). n=3 independent experiments. Data are represented as mean ± SEM and were analyzed using unpaired two-tailed Student's *t*-test. ****$p < 0.0001$. **c** Confocal images showing the effect of insulin treatment on Orai1 localization in HEK293FT cells expressing mCherry-Flag-tagged Orai1. Scale bar = 25 μm.

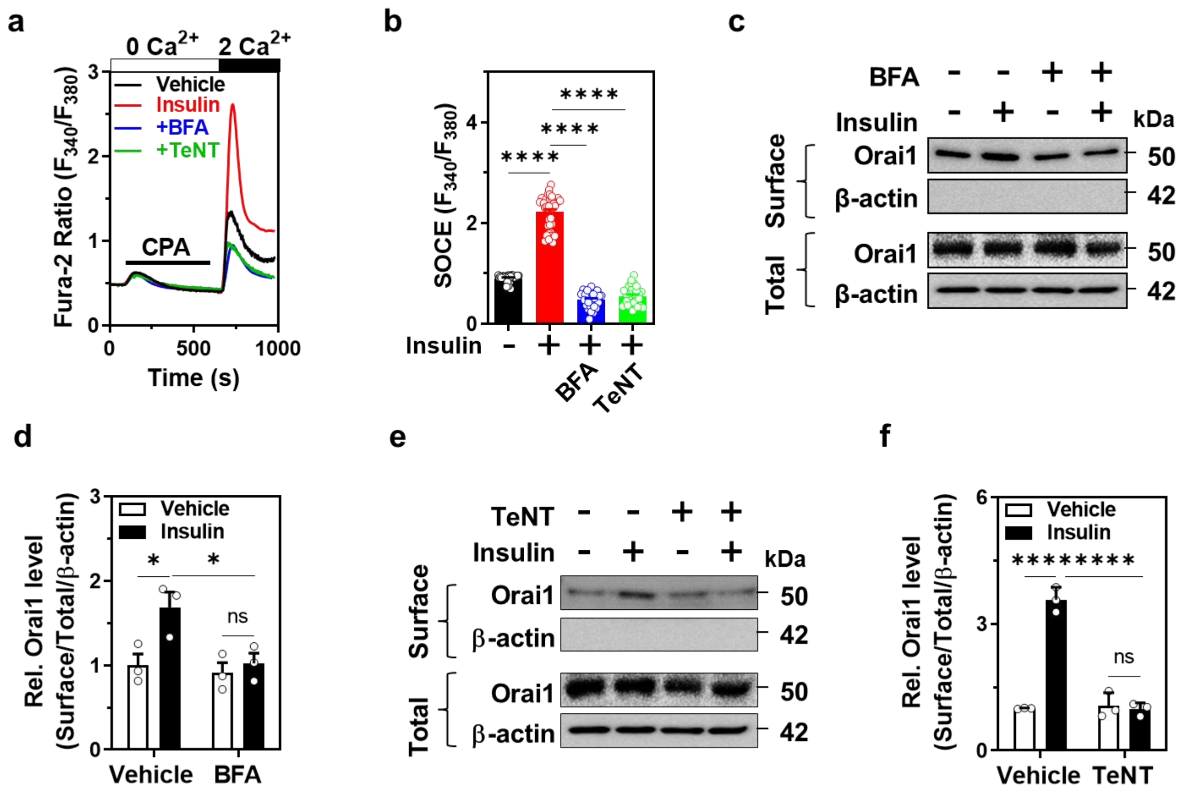

Supplementary Fig. 6 Insulin increases cell surface abundance of Orai1 by stimulating its exocytosis.

a Representative trace of insulin-stimulated store-operated Ca^{2+} entry **(**SOCE) in addition to brefeldin A (BFA, 10 µM, 8 hr) or tetanus toxin (TeNT, 60 nM, 8 hr). **b** Summary of the SOCE in panel (**a**). *n* = 26, 35, 29, 29 cells per each group. **c**-**f** Biotinylation assay showing the effects of BFA (**c**-**d**) and TeNT (**e**-**f**) treatment on cell surface abundance of Orai1 after insulin stimulation. All the experiments were repeated 3 times independently. Data are represented as mean ± SEM and were analyzed using one-way ANOVA followed by Tukey's multiple comparisons test (**b**, **d**, and **f**). $*p < 0.05$, $****p < 0.0001$; ns, not significant.

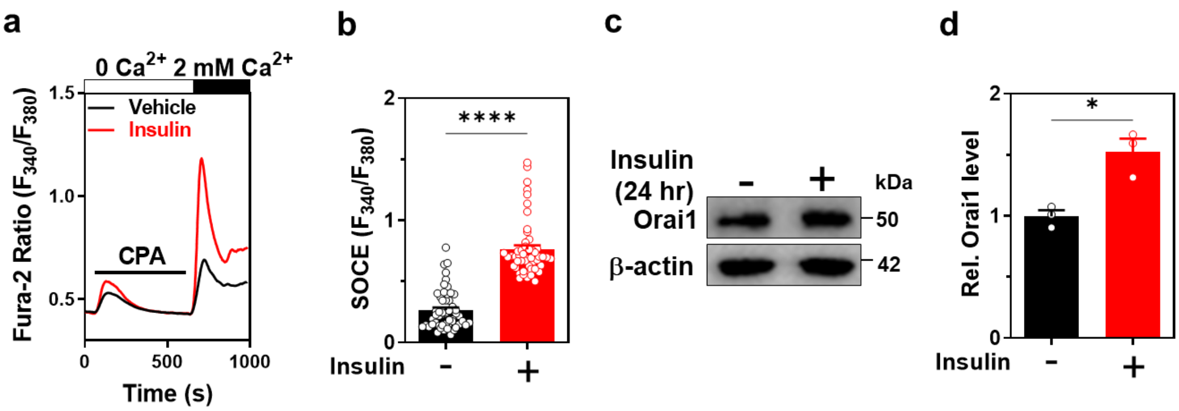

Supplementary Fig. 7 Effect of long-term incubation of insulin on store-operated Ca^{2+} entry (SOCE) and Orai1 protein expression in podocytes.

a Representative SOCE traces showing the effect of long-term insulin treatment (for 24 h) in cultured mouse podocytes. **b** Summary of the SOCE in panel (**a**). n = 53 (Vehicle) and 50 (Insulin) cells. **c** Immunoblotting showing the effect of long-term insulin treatment (for 24 h) on Orai1 expression in podocytes. **d** Summary of the relative (Rel.) Orai1 level in panel (**c**). n = 3 independent experiments. Data are represented as mean ± SEM and were analyzed using unpaired two-tailed Student's t-test in (**b** and **d**). $*p < 0.01$, $****p < 0.0001$.

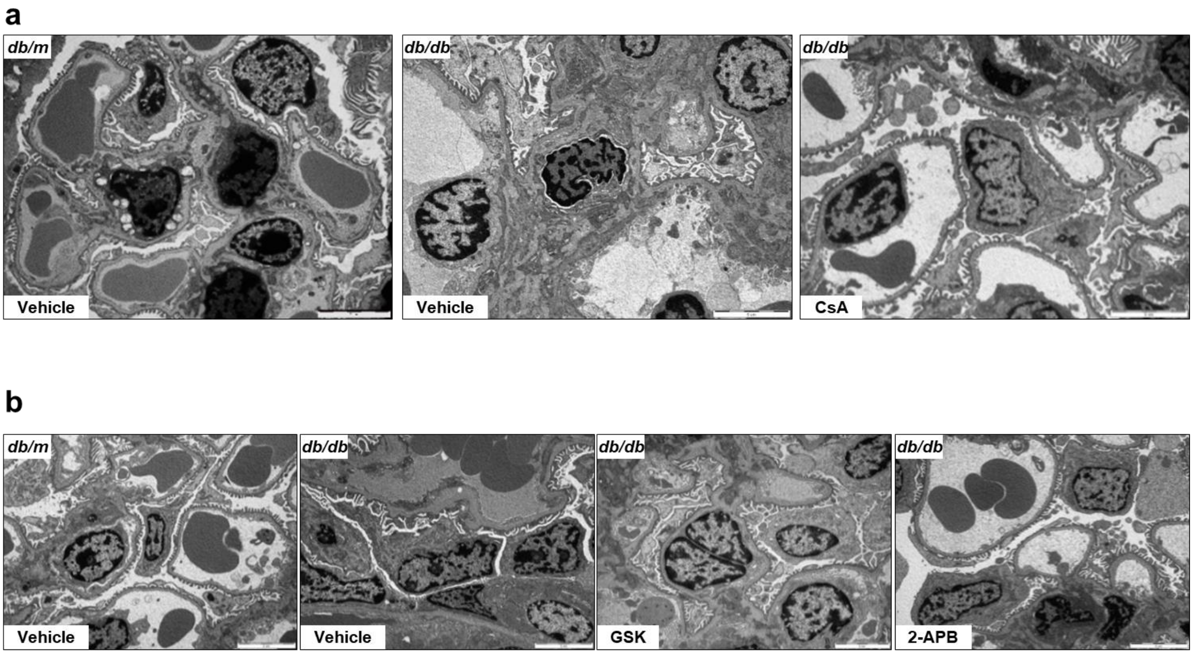

Supplementary Fig. 8 Representative TAM images in CSA, GSK-7975A, and 2-APB *i.p.* injected *db/db* mice

a-**b** Representative TEM images of kidney tissue in *db/m* and *db/db* mice with administration of CsA (**a**) or GSK or 2-APB (**b**). All images showed similar to the representative images of each group (n = 3 for *db/m*+Vehicle, *db/db*+Vehicle, *db/db*+CsA, *db/db*+GSK, *db/db*+2-APB) and were taken 5 different locations randomly in one mouse. Magnification is 5,000X. Scale bar = 5 μm.

Supplementary Table 1. List of mouse primer sequences used for PCR.

Gene (Accession no.)	Purpose	Primer sequence (5' to 3')		Size(bp)
Mouse 18s (NR_003278.1)	Quantitative real-time PCR	Forward	CGG CGT TAT TCC CAT GAC	108
		Reverse	GCC CTT CCG TCA ATT CCT	
Mouse Orai1 (NM_175423.3)	Quantitative real-time PCR	Forward	ATG GTA GCG ATG GTG GAA GT	122
		Reverse	CTG ATC ATG AGG GCA AAC AG	
Mouse Orai2 (NM_178751.3)	Quantitative real-time PCR	Forward	CTG CAT GAG ATC CAC CAC AC	97
		Reverse	GGG GAC AGG AGT ATG GGA TT	
Mouse Orai3 (NM_198424.3)	Quantitative real-time PCR	Forward	AAC CTC ACA CCA TCC TCT GC	96
		Reverse	GCC TGG TCC ATG AGC ACT AT	
Nphs2_Cre	Genotype	Forward	GCG GTC TGG CAG TAA AAA CTA TC	100
		Reverse	GTG AAA CAG CAT TGC TGT CAC TT	
Orai1_flox	Genotype	Forward	AGG CGG CCT ATA ATT CCA GCT TCA	WT;222 *Orai1*$^{fl/-}$;222,378 *Orai1*$^{fl/fl}$;378
		Reverse	AGG TGG ATG TTG CTG AGA GAC CAA	
International Positive control	Genotype	Forward	CTA GGC CAC AGA ATT GAA AGA TCT	324
		Reverse	GTA GGT GGA AAT TCT AGC ATC ATC C	
