# Supplementary material for: Insulin-activated store-operated Ca2+ entry via Orai1 induces podocyte actin remodeling and causes proteinuria
Source: Nat Commun. 2021 Nov 11;12:6537. doi: 10.1038/s41467-021-26900-w (PMC8586150; doi:10.1038/s41467-021-26900-w)
Supplement: Supplementary file 3 — Reporting Summary [file 41467_2021_26900_MOESM3_ESM.pdf]

nature portfolio

Corresponding author(s): DBPR NCOMMS-16-25536C

Last updated by author(s): Oct 22, 2021

Reporting Summary

Nature Portfolio wishes to improve the reproducibility of the work that we publish. This form provides structure for consistency and transparency in reporting. For further information on Nature Portfolio policies, see our Editorial Policies and the Editorial Policy Checklist.

Statistics

For all statistical analyses, confirm that the following items are present in the figure legend, table legend, main text, or Methods section.

n/a	Confirmed	
☐	☒	The exact sample size (*n*) for each experimental group/condition, given as a discrete number and unit of measurement
☐	☒	A statement on whether measurements were taken from distinct samples or whether the same sample was measured repeatedly
☐	☒	The statistical test(s) used AND whether they are one- or two-sided<br>*Only common tests should be described solely by name; describe more complex techniques in the Methods section.*
☒	☐	A description of all covariates tested
☐	☒	A description of any assumptions or corrections, such as tests of normality and adjustment for multiple comparisons
☐	☒	A full description of the statistical parameters including central tendency (e.g. means) or other basic estimates (e.g. regression coefficient) AND variation (e.g. standard deviation) or associated estimates of uncertainty (e.g. confidence intervals)
☐	☒	For null hypothesis testing, the test statistic (e.g. *F*, *t*, *r*) with confidence intervals, effect sizes, degrees of freedom and *P* value noted<br>*Give P values as exact values whenever suitable.*
☒	☐	For Bayesian analysis, information on the choice of priors and Markov chain Monte Carlo settings
☒	☐	For hierarchical and complex designs, identification of the appropriate level for tests and full reporting of outcomes
☒	☐	Estimates of effect sizes (e.g. Cohen's *d*, Pearson's *r*), indicating how they were calculated

Our web collection on statistics for biologists contains articles on many of the points above.

Software and code

Policy information about availability of computer code

Data collection	The MetaFluor software (Vesion 6.1, Sutter Instrument, Novato, CA, USA) for Ca2+ imaging, EPC-9 patch-clamp amplifier with the Pulse/ Pulsefit (v8.50) software (Heka Electronik, Lambrecht, Germany) for electrophysiological recording, a real-time PCR system (7900HT, Applied Biosystems, Foster City, CA, USA) for qPCR data, a laser scanning confocal microscope (TCS SPE, Leica Microsystems GmbH, Wetzlar, Germany; or LSM800, Zeiss, Jena, Germany) for immunofluorescence data.
Data analysis	GraphPad Prism software (version 9, GraphPad Software, San Diego, CA) for graphs and statistics, ZEN 2.3 software (Zeiss, Jena, Germany) for confocal data analysis, Image J 1.53c (NIH, Bethesda, Mayland, USA) for immunoblotting and densitometry analysis, the Pulse/Pulsefit (v8.50) software (Heka Electronik, Lambrecht, Germany) for patch clamp data analysis.

For manuscripts utilizing custom algorithms or software that are central to the research but not yet described in published literature, software must be made available to editors and reviewers. We strongly encourage code deposition in a community repository (e.g. GitHub). See the Nature Portfolio guidelines for submitting code & software for further information.

Data

Policy information about availability of data

All manuscripts must include a data availability statement. This statement should provide the following information, where applicable:

- Accession codes, unique identifiers, or web links for publicly available datasets
- A description of any restrictions on data availability
- For clinical datasets or third party data, please ensure that the statement adheres to our policy

The authors declare that all data supporting the findings of this study are available within the paper, Supplementary Information files, and the Source Data files. Source Data files including original data of Ca2+ imaging, animal experiment and western blot have been deposited in Figshare.com (DOI: 10.6084/

m9.figshare.16618213). All other data supporting the findings of this study are available from the corresponding author on a reasonable request. Source data are also provided as a Source Data file with this paper.

Field-specific reporting

Please select the one below that is the best fit for your research. If you are not sure, read the appropriate sections before making your selection.

☒ Life sciences ☐ Behavioural & social sciences ☐ Ecological, evolutionary & environmental sciences

For a reference copy of the document with all sections, see nature.com/documents/nr-reporting-summary-flat.pdf

Life sciences study design

All studies must disclose on these points even when the disclosure is negative.

Sample size	Sample sizes for in vitro experiments were chosen based on the standard practices of the field. At least 3 independent experimental replicates were performed for all cases. For in vivo experiments, we used at least 5 mice per group in order to have a power of 0.8 and a probability of type I error of 0.5 or less (Srikanth et al., Nat Immunol:20:112-20, 2019).
Data exclusions	No data were excluded from the experiments.
Replication	Experiments were repeated at least three times with similar results to ensure reproducibility of data.
Randomization	Knockout mice were allocated into wild-type, hetero and KO group based on genotyping. Mice (db/db and db/m) used in albuminuria studies were randomized. Samples for histochemistry and electron microscopy from mice were analyzed by a renal pathologist in a blinded manner.
Blinding	The TEM images were assessed by a renal pathologist in a blinded fashion. The investigators were blinded to group allocation during data collection and analysis.

Reporting for specific materials, systems and methods

We require information from authors about some types of materials, experimental systems and methods used in many studies. Here, indicate whether each material, system or method listed is relevant to your study. If you are not sure if a list item applies to your research, read the appropriate section before selecting a response.

Materials & experimental systems

n/a	Involved in the study	
☐	☒	Antibodies
☐	☒	Eukaryotic cell lines
☒	☐	Palaeontology and archaeology
☐	☒	Animals and other organisms
☒	☐	Human research participants
☒	☐	Clinical data
☒	☐	Dual use research of concern

Methods

n/a	Involved in the study	
☒	☐	ChIP-seq
☒	☐	Flow cytometry
☒	☐	MRI-based neuroimaging

Antibodies

Antibodies used	Primary antibodies for VAMP2 (1:1000 dilution, ab181869), Orai1 (1:1000 dilution, ab86748), and b-actin (1:5000 dilution, ab6276) were obtained from Abcam. STIM1 (1:1000 dilution, 610954) and Flag-HRP (1:5000 dilution, A8592) were purchased from BD Biosciences (Clontech, Palo Alto, CA, USA) and Sigma-Aldrich, respectively. Antibodies against Akt (1:2000 dilution, 9272), p-AktSer473 (1:2000 dilution, 9271), and p-AktThr308 (1:2000 dilution, 2965) were obtained from Cell Signaling Technology (Beverly, MA, USA). GAPDH (1:10000 dilution, sc25778), and antibodies against synaptopodin (1:1000 dilution, sc21537) were purchased from Santa Cruz Biotechnology. Antibodies against Orai2 (1:1000 dilution, ACC-061), Orai3 (1:1000 dilution, ACC-065), STIM2 (1:100 dilution, ACC-064) and TRPC6 (1:500 dilution, ACC-120) were obtained from Alomone Labs (Jerusalem, Israel). For immunofluorescence experiments, goat anti-synaptopodin (1:40 dilution; sc21537, Santa Cruz Biotechnology), rabbit anti-Orai1 (1:100 dilution, NBP1-46470, Novus, Littleton, CO, USA), rabbit anti-Flag-tag (1:20 dilution; A00170, GenScript), anti-goat-Alexa 488 (1:200 dilution; A11001, Life Technology, Eugene, OR, USA), anti-goat-Cy3 (1:200 dilution; 705-165-003, Jackson ImmunoResearch), and anti-rabbit-Alexa 594 (1:200 dilution; A31631, Life Technology, Eugene, OR, USA), rabbit anti-Orai1 (1:100 dilution, ab86748, Abcam, Cambridge, MA, US), goat anti-nephrin (1:50, AF3159, R&D systems), goat anti-synaptopodin (1:40 dilution, sc21537), or rabbit anti-paxillin (1:250 dilution, 1500-1, Epitomics, Burlingame, CA, USA), with a secondary antibody, either anti-rabbit-Alexa-594 (1:200 dilution, A-31631, Life Technology), anti-goat-Alexa-488 (1:200 dilution, A11078, Life Technology), or anti-rabbit-Alexa-488 (1:200 dilution, A31627, Life Technology) .
Validation	Antibodies are already validated based on manufacturer's data and their widespread use in multiple published article. Additionally,

Validation

some of antibodies were validated ourselves using siRNA knockdown, plasmid overexpression and positive and negative control sample. Orai1 (rabbit polyclonal, abcam, ab86748, https://www.abcam.com/orai1-antibody-ab86748.html); Orai1 (rabbit polyclonal, Novus, NBP1-46470, https://www.novusbio.com/products/orai1-antibody_nbp1-77289); Orai2 (rabbit polyclonal, alomone labs, ACC-061, https://www.alomone.com/p/anti-orai2/ACC-061); Orai3 (rabbit polyclonal, alomone labs, ACC-065, https://www.alomone.com/p/anti-orai3/ACC-065); STIM1 (mouse monoclonal, BD science, 610954, https://www.bdbiosciences.com/en-us/products/reagents/microscopy-imaging-reagents/immunofluorescence-reagents/purified-mouse-anti-gok-stim1.610954); STIM2 (rabbit polyclonal, alomone labs, ACC-064, https://www.alomone.com/p/anti-stim2/ACC-064); TRPC6 (rabbit polyclonal, alomone labs, ACC-120, https://www.alomone.com/?s=ACC-120&submit=Search); VAMP2 (rabbit monoclonal, abcam, ab181869, https://www.abcam.com/vamp2-antibody-epr12790-ab181869.html); β-actin (mouse monoclonal, abcam, ab6276, https://www.abcam.com/beta-actin-antibody-ac-15-ab6276.html); GAPDH (rabbit polyclonal, Santa Cruz Biotechnology, Inc., sc25778, https://datasheets.scbt.com/sc-25778.pdf); Flag-HRP (monoclonal produced in mouse, Sigma-Aldrich, A8592, https://www.sigmaaldrich.com/CH/en/sds/sigma/a8592); Akt (rabbit polyclonal, Cell Signaling, 9272, https://www.cellsignal.com/products/primary-antibodies/akt-antibody/9272); p-AktSer473 (rabbit polyclonal, Cell Signaling, 9271, https://www.cellsignal.com/products/primary-antibodies/phospho-akt-ser473-antibody/9271); p-AktThr308 (rabbit monoclonal, Cell Signaling, 2965, https://www.cellsignal.com/products/primary-antibodies/phospho-akt-thr308-c31e5e-rabbit-mab/2965); synaptopodin (goat polyclonal Santa Cruz Biotechnology, Inc., sc21537, https://datasheets.scbt.com/sc-21537.pdf); Nephrin (goat polyclonal, R&D Systems, AF3159, https://www.rndsystems.com/products/mouse-nephrin-antibody_af3159); Paxillin (rabbit monoclonal, abcam, ab32084, https://www.abcam.com/paxillin-antibody-y113-ab32084.html); Alexa Fluor™ 594 Phalloidin (Invitrogen, A12381, https://www.thermofisher.com/order/catalog/product/A12381); Rabbit anti-Flag-tag (GenScript, A00170, https://www.genscript.com/antibody/A00170-DYKDDDDK_tag_Antibody_pAb_Rabbit.html); Donkey anti-Goat IgG Alexa 488 (polyclonal, Invitrogen, A-11055, https://www.thermofisher.com); Donkey anti-Rabbit IgG Alexa 488 (polyclonal, Jackson ImmunoResearch, 711-545-152, https://www.jacksonimmuno.com/catalog/products/711-545-152); Cy™3 AffiniPure Donkey Anti-Goat IgG (polyclonal, Jackson ImmunoResearch, 705-165-003, https://www.jacksonimmuno.com/catalog/products/705-165-003); Donkey anti-rabbit IgG Alexa 594 (polyclonal, Invitrogen, A-21207, https://www.thermofisher.com/order/genome-database/dataSheetPdf?producttype=antibody&productsubtype=antibody_secondary&productId=A-21207&version=171).

Eukaryotic cell lines

Policy information about cell lines

Cell line source(s)

HEK293FT cell line were obtained from American Type Culture Collection center (ATCC, Manassas, VA). The immortalized mouse podocyte cell line was a kindly provided from Dr. Peter Mundel (Harvard Medical School, USA) as described in previous our paper (Kim et al., J Am Soc Nephrol 28:140-51, 2016).

Authentication

None of the cell lines used were authenticated.

Mycoplasma contamination

All cell lines were tested for mycoplasma routinely using a PCR-based method. All cell lines used were contamination free.

Commonly misidentified lines
(See ICLAC register)

None of misidentified cell lines were used in this study.

Animals and other organisms

Policy information about studies involving animals; ARRIVE guidelines recommended for reporting animal research

Laboratory animals

To generate conditional Orai1 knockout (KO) mice, mice homozygous for the Orai1 allele flanked by the loxP site (Orai1fl/fl) were backcrossed to C57/BL6/J mice for at least ten generations. Orai1fl/- mice and Podocin-cre (Nphs2.Cre+ = B6.Cg-Tg(NPHS2-cre)295Lbh/J) mice were generated as described previously (Srikanth et al., Nat Immunol, 20:152-62, 2019; Gee et al., Nat Commun, 7:10822, 2016). Trpc6-/- mice have been described previously (Xie et al., Nat Commun 3:1238, 2012). Six-week-old male BKS.Cg-m+/+Leprdb/BomTac db/m and db/db mice were purchased from Taconic Farms (Germantown, NY, USA); db/m mice were used as controls in all experiments. All mice were raised in the individual ventilated cage (IVC) racks at a constant temperature (22 ± 3 °C) and constant relative humidity (50 ± 10%) using fluorescent lamps (lights are on 6:00 to 18:00) for 12 h and fed with solid feed 5L79® (LabDiet, St. Louis, MO, USA). All mice were maintained in pathogen-free barrier facilities.

Wild animals

No wild animals were used in this study.

Field-collected samples

This study did not involve samples collected from field.

Ethics oversight

All experimental protocols involving mice were approved by the Yonsei University Wonju College of Medicine Institutional Animal Care and Use Committee (YWC-170919-1, YWC-130826-2, and YWC-161222-1).

Note that full information on the approval of the study protocol must also be provided in the manuscript.
